# Cancer care delivery innovations, experiences and challenges during the COVID-19 pandemic: The Rwanda experience

**DOI:** 10.7189/jogh.11.03067

**Published:** 2021-04-17

**Authors:** Grace Umutesi, Cyprien Shyirambere, Jean Bosco Bigirimana, Sandra Urusaro, Francois Regis Uwizeye, Evrard Nahimana, Jean D’Amour Tuyishimire, Pacifique Mugenzi, Joel M Mubiligi, Francois Uwinkindi, Fredrick Kateera

**Affiliations:** 1 Partners in Health/ Inshuti Mu Buzima, Rwinkwavu, Rwanda; 2 Department of Global Health, University of Washington, Seattle, Washington, USA; 3 Zipline-Rwanda, Muhanga/Kayonza Distribution Centers, Muhanga, Rwanda; 4 Rwanda Cancer Center, Rwanda Military Hospital, Kigali, Rwanda; 5 Rwanda Biomedical Center, Ministry of Health, Kigali, Rwanda

Globally, cancer is the second leading cause of mortality. In 2018, 9.6 million lives were lost to cancer of which over 70% occurred in low and middle-income countries (LMICs) where limited access to cancer care and overwhelming late disease presentations negatively impact cancer related survival and quality of life [1]. Moreover, globally, new cancer cases are expected to increase from 18.1 million in 2018 to 21.4 million by 2030 [2]. In settings of poor health care systems and impoverished communities, the scarcity of and limited access to diagnostic and treatment modalities negatively impacts health outcomes and undermines achievement of the universal health care coverage (UHC) targets.

Over the past 20 years, Rwanda has recorded gains in key health indicators including increased life expectancy (from 48.6 in 2000 to 67.4 in 2015); declines in maternal mortality (from 1071 in 2000 to 210 per 100 000 live births in 2015) [3]. Concurrently, impressive gains were registered in the control of infectious diseases such as HIV, tuberculosis and malaria [3]. However, little gains have been recorded for the management of non-communicable diseases (NCDs) where age-standardized NCD mortality rates slightly decreased from 894.9 to 548.6 deaths per 100 000 people from 2000 to 2016 [4,5]. Anecdotally, plausible hindrances to the prevention and control of NCDs in Rwanda include low community awareness, lack of trained providers, limited access to diagnostic services and treatment capacity for complicated cases [5].

## CANCER CARE IN RWANDA

In July 2012, the Rwandan Ministry of Health (RMoH) in collaboration with Partners in Health/ Inshuti Mu Buzima (PIH/ IMB) and other partners launched the Butaro Cancer Center of Excellence (BCCOE) in rural Northern Rwanda. BCCOE provides cancer related diagnostic, chemotherapy, palliative and surgery – such as mastectomies – services [6]. To optimize patient outcomes and access to other complementary services, BCCOE partners with national and regional referral hospitals for advanced cancer surgeries and radiotherapy care. Because the majority of patients are poor, BCCOE offers complementary social support (meals and transportation) and an oncology patient support center for accommodation and psychological support. Since its operationalization, BCCOE has served patients from all Rwanda and neighboring countries such as Burundi and the Democratic Republic of Congo (DRC). By the end of 2019, BCCOE had treated over 11 116 cancer patients, of whom 94.2% were adults, among them, 74.7% were women. The most common adult malignances were breast (13.7%), cervix (11.4%) and cancers of the gastrointestinal tract.

The increase in cancer case incidence in Rwanda from 1828 in 2012 to 2933 cases in 2018 calls for expansion of cancer care services [7]. In February 2020, the Rwanda Cancer Center (RCC) hosted at the Rwanda Military Hospital (RMH) in Kigali was launched and since then, RCC has provided radiotherapy services to over 696 patients and it is currently fast-tracking trainings and necessary set-ups to provide chemotherapy services [8]. The current radiation equipment at RCC provides advanced-beam radiation using Volumetric Modulated Arc Therapy(VMAT) and brachytherapy, a preferable treatment modality for patients with potentially curable cancers is not yet available in Rwanda. Patients requiring brachytherapy services – hitherto accessed through Nairobi Hospital in Kenya – ceased with the COVID-19 travel restrictions between countries.

## CANCER CARE DURING THE COVID-19 PANDEMIC

The first COVID-19 case in Rwanda was confirmed on March 14, 2020. A week later, March 21, 2020, an abrupt nation-wide lock-down initially for a 2-week period that later was extended by a month was instituted to curtail COVID-19 spread. In consequence, immediate cessation of key social, economic, health care and education activities happened [9]. Among cancer patients treated at BCCOE, routine follow-up visits, drug refills, access to diagnostic and treatment services all momentarily ceased. Given the unpredictability of the COVID-19 pandemic, on-going and future response strategies will continue to affect access to and use of health care services for patients in rural communities.

## AD HOC RESPONSIVE CANCER CARE INNOVATIONS

PIH/IMB collaborated with the RMoH, RCC/RMH, National Police, Ministry of local government and private for profit Organizations (Zipline) to implement innovative interventions to ensure available and continued health care services. Four main interventions were undertaken between end March and June 2020 to: 1) ensure access to RCC and BCCOE for continued cancer care; 2) set up a drone-based drug re-fill community delivery system; 3) support continued cancer care among BCCOE enrolled patients living in DRC and Burundi; and 4) provide social economic support to vulnerable oncology patients. A number of factors influenced the design and implementation of these innovations including but not limited to: a) treatment (whether the patient was on oral vs intravenous chemotherapy or needed radiation therapy); b) accessibility of the patients to either RCC or BCCOE; c) severity of the disease (palliative vs curative cases); d) availability of core infrastructure and space at RMH and e) lifting of key restrictions like transport routes.

**Figure:**
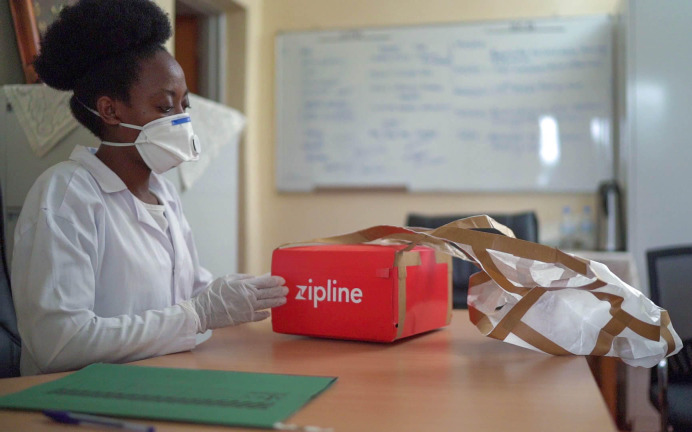
Photo: Nurse at Kirehe PIH office receiving a Zipline package of cancer oral medicine for a patient. Kirehe, April 14, 2020. Photo credit: Pacifique Munemana for PIH/IMB.

In partnership with the RMH, PIH/IMB supported RCC to provide chemotherapy (oral and intravenous) for patients located in the central Kigali, Southern and Eastern provinces as well as provision of radiation to all patients from all provinces. The first patient in this initiative was treated at RCC on March 30 2020. To facilitate this, PIH/IMB availed; 1) vehicles for pick-ups and drop-offs of patients between RMH and their homes or nearest places; 2) cancer care experienced human resource team of one physicians and two nurses from the BCCOE; 3) drugs, supplies, all required complementary clinical evaluations and tests; 4) a coordination team of two patients navigators, a project coordinator and the PIH/IMB oncology program manager to provided logistics support (scheduling patients appointments, communicating with patients, arranging transport and accommodation); 5) in Kigali, a residential facility to provide accommodation, meals and transport support for out of town patients; and 6) transport facilitation for patients from the northern and southern provinces to access care at the BCCOE. Concurrently, RMH availed 2 oncologists and the RCC director to support the expedited operationalization of the infusion center at the RCC. A weekly patient list generated from OpenMRS (the electronic patient record system at BCCOE) by the PIH/IMB oncology team was used to plan for patient follow-up visits. Patients requiring a medical consultation or chemotherapy infusion accessed care either by being car-picked up from their homes by a PIH/IMB car the day before their scheduled clinic visit or via district hospitals’ (DH) referral in an ambulance. Patients referred from DHs had their pre-chemotherapy laboratory tests done at the DHs and the results were shared with an oncologist at the RCC or BCCOE before transporting the patient to their respective treatment sites. For patients transported to RCC, PIH/IMB availed accommodation and meals for an average of 45 patients per night and a nurse who monitored their well-being during their stay in Kigali. At the BCCOE, PIH/ IMB supported the provision of logistic support (transport, meals and accommodation) for 935 patients and 815 of these patients were hosted at the oncology patients’ support center in Butaro. Patients stayed in Kigali or Butaro for a maximum of 3 days and for efficiency, the logistics team scheduled appointments for patients from the same region.

Since its launch up to June 30 2020, the RCC chemotherapy infusion clinic had seen 378 patients over 815 visits with 73.6% of patients coming from outside Kigali City. The main cancer diagnoses treated at RCC were breast cancer (38.6%), cervical cancer (10.3%), and chronic myeloid leukemia (6.3%). Of the 378 patients treated at RCC, 64% patients received chemotherapy infusions, 17.5% received oral chemotherapy while the rest were either physiologically unstable for chemotherapy or were referred for other procedures at other health facilities. Concurrently, at BCCOE, 1982 visits were performed for 935 patients; among which 55% patients received IV chemotherapy during the lockdown period. The two centers supported care for 1313 patients among which 756 patients had IV chemotherapy (68% patients at BCCOE and 32% patients at RCC), and 63 patients were referred from BCCOE for radiotherapy at RCC.

In collaboration with Zipline – a for profit drone based delivery organization, drone deliveries were deployed for oral drug drops for patients living in remote settings and who did not need a clinical review. To achieve this, the PIH/IMB oncology and Zipline teams defined patient locations, designed flight routes and delivered oral medications at the nearest and most accessible HCs and DHs. This innovative approach was complemented with virtual clinics where clinicians consulted patients on the phone and ordered necessary laboratory tests remotely at DHs closest to the patient to ensure safe administration of medications. The index Zipline pill drop was done on 14 April 2020. A total of 73 patients on oral chemotherapy were assessed remotely by a clinician and received their oral therapy dropped off at the nearest health centers (HCs) either through Zipline’s drones or via PIH/IMB organized car drop-offs at HCs near road networks. These approaches improved the treatment experience of cancer patients who previously had to travel long distances to get their medical refills.

Two different approaches were used to support cancer care for patients from DRC and Burundi. Patients from the DRC were contacted and asked to travel to a border point Rwanda were they were picked up from the border after getting the clearance from the immigration team and driven to BCCOE by a PIH/ IMB car where they were provided a space to quarantine. Additional support included psychosocial support and meals while at BCCOE as well as two COVID-19 RT-PCR tests as recommended by the RMOH. Regarding Burundian patients who could not travel to Rwanda, the PIH/IMB team worked with an International Courier (DHL) to send oral drugs to patients in Burundi. Within Burundi, PIH/IMB collaborated with the Burundi Cancer patient’s association who receive the medications, reached out to patients and facilitated drug distribution. Between May and December 2020, four separate couriers were completed, the first and last batches arriving Burundi on the May 8, 2020 and December 10, 2020, respectively. These interventions allowed us to provide uninterrupted care to 93 patients who were outside the country where 45 cancer patients (35 from Burundi and 10 from DRC) received their oral cancer medications while 48 patients from DRC were able to get their IV chemotherapy at BCCOE.

PIH/IMB availed social-economic support to the most vulnerable patients enrolled in chronic diseases care programs. Among oncology patients, this support was extended to all patients on chemotherapy (oral or IV) and those belonging in the ubudehe category I or II. Ubudehe is a Rwandan home-grown household based socio-economic categorization mechanism for determining eligibility for Rwanda’s key social protection interventions [10]. Over a 2-month period, selected patients residing in the three PIH/ IMB supported districts of Kirehe, Kayonza and Burera were contacted by phone and invited to pick up a package at their respective HCs. The package included nutritional, hygienic and infection control household items. Among eligible patients who lived outside PIH/ IMB catchment area; direct cash transfers - equivalent to the cost of packages given to patients in the three PIH/IMB supported districts - via phone based mobile money solutions was done. In Total 155 and 413 patients from PIH/IMB and from 28 different non PIH/IMB supported districts, respectively, received social-economical packages.

## COLLABORATORS

To achieve these mile stones, PIH/IMB – an NGO that is built on an ethos of being nimble and responsive in accompaniment with the government catalyzed a partnership that included clinicians (PM, CS), program managers (GU, SU, JB, FRU), pharmacy and logistics experts (JT), national program leaders (FU) and global health leaders (EN, JM, FK). Collectively, we were all united to ensure a quick, collaborative, nimble and contextually relevant innovative solution to delivering cancer care during a pandemic with all associated limitations in access to life saving services. This success also underlines good will from political and national leadership and a collaborative spirit needed to design and implement innovative adaptive public health responses.

## CHALLENGES EXPERIENCED

We experienced multiple challenges. First, due to the COVID-19 related restrictions in movements, a minority of people were transported to BCCOE at any given time. This led to running multiple trips across the entire country to bring in and back the patients. Additionally, the risks of infections while travelling for cancer patients required to have at least one nurse to accompany patients on all trips. Second, limitations in cancer expertise, chemotherapy and care management protocols at RCC, additional skilled human resource to provide the needed support were drafted in from BCCOE. Third, with only 67% mobile phone penetration in 2017, on multiple occasions, the team could not reach patients or their next of kin to deliver of their social economic support packages [11]. However, leveraging the structures of CHWs, we were able to reach over 95% of our patients who could not be reached directly by phone. Even when oral therapies were delivered at HCs by cars or drones, the travel distance to HCs were, for some patients, long and could only be accessed by walking thus complicating access to these drugs. Fourthly, our efforts focused on patients needing chemotherapy or radiotherapy for curative intent; thus, palliative cases were largely not supported due to the transport difficulties and concerns of exposure to COVID-19 risks including death.

## HEALTH SERVICE DELIVERY LESSONS LEARNED

The robustness of a health system is tested in times of crisis. The adhoc measures we instituted were possible largely because of an existing robust national health care system. Additionally, the support received from key government institutions reflects a government that is nimble, responsive to real needs and works with different fostered program implementation during challenging times. This was demonstrated by the positive and quick response of the RMOH, Rwanda National Police and Local Government to authorize our team to implement these activities during the strict lockdown period. In addition, the strong network of community health care workers (CHWs) allowed us to connect with patients who could not be reached on phone. A number of key lessons were learned. First, to achieve UHC, it is crucial to prioritize strengthening health systems to more effectively support chronic care service delivery. Second, especially in epidemics, a combination of nimble systems, National leadership and strong partnerships is essential for quick and effective disease focused global health care responses. Third, a robust and inter-hospital connected electronic medical records is a strategic and vitally contributory tool for a strong coordinated decentralized system to effectively support accessible patient care services. Fourthly, especially in developing settings where health care expertise remains low, developing disease management protocols for co-shared patient care will be vital to optimize patient outcomes and set-up of effective collaborative follow-up programs. Fifth, traditionally doctor-led health care systems need to be replaced with team-based approaches with strong task-shifting mechanisms. These approaches should be paired with a more patient-centered comprehensive care package that includes social support, financing support, patient education, and family engagement to optimize impact where patients are constrained by other structural and system limitations. Ultimately, these approaches will have ripple effect in health care delivery in Rwanda and similar settings.
